# Cephalosporin-Glycopeptide Combinations for Use against Clinical Methicillin-Resistant *Staphylococcus aureus* Isolates: Enhanced *In vitro* Antibacterial Activity

**DOI:** 10.3389/fmicb.2017.00884

**Published:** 2017-05-18

**Authors:** Hung-Jen Tang, Chih-Cheng Lai, Chi-Chung Chen, Chun-Cheng Zhang, Tzu-Chieh Weng, Wen-Liang Yu, Hung-Jui Chen, Yu-Hsin Chiu, Wen-Chien Ko, Yin-Ching Chuang

**Affiliations:** ^1^Department of Medicine, Chi Mei Medical CenterTainan, Taiwan; ^2^Department of Health and Nutrition, Chia Nan University of Pharmacy and ScienceTainan, Taiwan; ^3^Department of Intensive Care Medicine, Chi Mei Hospital—Liou YingTainan, Taiwan; ^4^Medical Research, Chi Mei Medical CenterTainan, Taiwan; ^5^Medicine, Chi Mei Hospital—Liou YingTainan, Taiwan; ^6^Department of Internal Medicine and Center of Infection Control, National Cheng Kung University HospitalTainan, Taiwan; ^7^Department of Medicine, College of Medicine, National Cheng Kung UniversityTainan, Taiwan

**Keywords:** glycopeptides, cefazolin, cefmetazole, cefotaxime, cefepime, combination therapy, synergism, MRSA

## Abstract

The empirical combination of both a beta-lactam and glycopeptide to counter potential staphylococcal pathogens may improve the clinical outcomes for cases of *Staphylococcus aureus* bacteremia. We reported comparative *in vitro* studies of combination effects of different cephalosporins (i.e., cefazolin, cefmetazole, cefotaxime, and cefepime) combined with glycopeptides for 34 randomly selected methicillin-resistant *S. aureus* (MRSA) isolates by three methods, including the checkerboard, time-killing, and combination MIC measurement methods. Thirteen SCC*mec* type III isolates with a cefazolin MIC of ≥ 128 μg/mL were classified as the high-cefazolin MIC (HCM) group, whereas 13 SCC*mec* type IV and 8 SCC*mec* type V isolates were classified as the low-cefazolin MIC (LCM) group. With the checkerboard method, synergism was present for vancomycin-based combinations at 30.8–69.2 and 13.6–66.7%, as well as teicoplanin-based combinations of 38.5–84.6 and 0–47.6%, of the HCM and LCM isolates, respectively. No antagonism was noted. The *in vitro* inhibitory activity was evident even at a low concentration of 1/512x MIC of cephalosporin combined with sub-inhibitory concentrations (1/2x MIC) of a glycopeptide. With time-killing assays, synergism was noted at 1/2x or 1x susceptible breakpoint concentrations (SBCs) of a cephalosporin combined with 1/4 or 1/2 MIC of a glycopeptide. In the presence of 1/2 SBC of a cephalosporin, vancomycin or teicoplanin MICs decreased an average of 2.0- to 6.6- or 1.6- to 5.5-fold, respectively. With 8 μg/mL cephalosporin, the decline of glycopeptide MICs was most obvious in the presence of cefmetazole. In conclusion, cephalosporin-glycopeptide combinations at clinically achievable concentrations can exhibit *in vitro* synergistic antibacterial activity against clinical MRSA isolates. Such combinations require more clinical data to support their application for use in human MRSA infections.

## Introduction

Methicillin-resistant *Staphylococcus aureus* (MRSA) is a not only nosocomial, it is also a community pathogen that can cause a variety of infections, leading to significant morbidity and mortality (Ray et al., 2011; Khokhlova et al., 2015). Beyond its high virulence, MRSA is also notorious for its emerging vancomycin resistance (Lai et al., 2015; Teh et al., 2015). Recently, there has been increasing evidence supporting the poor efficacy of vancomycin in treating MRSA infections due to rising vancomycin MICs in clinical MRSA isolates, increasing from 0.25 to 2.0 μg/mL (Hawser et al., 2011; Chang et al., 2015; Niveditha and Sujatha, 2015). Therefore, several combination regimens, including rifampicin- or fosfomycin-based combinations, have been proposed to overcome the therapeutic disadvantage of vancomycin (Perlroth et al., 2008; Tang et al., 2011, 2012, 2013).

Previous studies suggested that the empirical combination of a beta-lactam and anti-MRSA agent to counter potential staphylococcal pathogens (MSSA and MRSA) may improve the clinical outcome (Lodise et al., 2007; Mongkolrattanothai et al., 2009). The concept that combinations of vancomycin and beta-lactams can be synergistic against staphylococci with reduced susceptibilities to vancomycin was mentioned more than a decade ago (Climo et al., 1999). An *in vitro* pharmacodynamic study of vancomycin alone, cefazolin alone or in combination against MRSA was recently reported (Hagihara et al., 2012). The synergism of cefepime or cefpirome combined with vancomycin or teicoplanin against MRSA isolates has also been explored (Carricajo et al., 2001; Lozniewski et al., 2001; Toyokawa et al., 2003). Here, we conducted a comparative *in vitro* study of the combination of any of four generation cephalosporins with either vancomycin or teicoplanin against MRSA isolates by three laboratory methods to elucidate the variation in the antibacterial activity of different cephalosporin-glycopeptide (C-G) combinations.

## Materials and methods

### Bacterial isolates

Thirty-four clinical MRSA isolates were randomly selected from the TIST study that collected clinical isolates from 22 hospitals between 2006 and 2010 (Hsueh, 2008). Staphylococci were identified by the colonial morphology, Gram stain, and coagulase test. MRSA isolates were further confirmed by the tube coagulase test and their growth on 6 μg/mL oxacillin salt agar screen plates. Isolates were stored at −70°C in Protect Bacterial Preservers (Technical Service Consultants Limited, Heywood, UK) until use. Their genetic relatedness was examined by pulse-field gel electrophoresis (PFGE) as previously described (Tenover et al., 1995; Lai et al., 2017).

### Antibiotics and MIC measurement

The tested antibiotics included oxacillin, erythromycin, gentamicin, clindamycin, rifampin, minocycline, cefazolin, cefmetazole, cefotaxime, cefepime, vancomycin (Sigma, St Louis, MO), fosfomycin (Ercros, Barcelona, Spain), linezolid, tigecycline (Pfizer, New York, NY), fusidic acid (Leo Pharma, Ballerup, Denmark), teicoplanin (Sanofi-Aventis, Bridgewater, NJ), ciprofloxacin (Bayer, Leverkusen, Germany), and daptomycin (Cubist Pharmaceuticals, Lexington, MA). The MIC determination by the agar dilution method and interpretation criteria were based on the recommendations of the Clinical and Laboratory Standards Institute (CLSI), Food and Drug Administration (FDA), and British Society for Antimicrobial Chemotherapy (Andrews, 2001; Clinical and Laboratory Standards Institute, 2012). For fosfomycin susceptibility tests, glucose-6-phosphate (25 μg/mL) was added to the agar plates. Daptomycin MIC was studied in Mueller-Hinton broth that was adjusted to 50 μg/mL calcium. *S. aureus* ATCC 29213 was used as a control strain in each run of MIC measurements. According to cefazolin MIC, MRSA were categorized into two groups, the high-cefazolin MIC (HCM, MIC > 128 μg/mL) and low-cefazolin MIC (LCM ≤ 128 μg/mL) groups.

### Panton-valentine leukocidin (PVL) and SCC*mec* genes

Genomic DNA from MRSA isolates was purified and used as a template for PCR amplification. PCR amplification of PVL genes, i.e., *lukF-PV* and *lukS-PV*, was performed as previously described (Montanaro et al., 2016). The primer sequences for the PVL genes were *luk-PV-1* and *luk-PV-2*. SCC*mec* typing was tested by multiplex PCR (M-PCR) according to a previously published protocol (Zhang et al., 2005). The M-PCR assay used 4 primers for *mec* (mecI-F, mecI-R, IS1272-F, and mecR1-R) and *ccr* (ccrAB-α2, ccrAB-α3, ccrAB-α4, and ccrAB-β2) complexes. A single-target PCR was used to detect type 5 *ccr* by ccrC-F and ccrC-R primers.

### Checkerboard method

To evaluate the *in vitro* effect of G/C combinations, the microdilution checkerboard method was used to calculate the fractional inhibitory concentrations (FICs), as recommended by the CLSI (Clinical and Laboratory Standards Institute, 2012; Lai et al., 2016). The following formulas were used to calculate the FIC index, which equals the FIC of drug A (MIC of drug A in combination/MIC of drug A alone) + FIC of drug B (MIC of drug B in combination/MIC of drug B alone). Synergism was defined as a FIC index of ≤ 0.5, an indifference FIC index of >0.5 but ≤ 4, and an antagonism FIC index of >4 (Lai et al., 2016).

### Time-kill method

Two MRSA isolates were randomly selected for another *in vitro* measurement of the inhibitory effect of combination regimens, as recommended by the CLSI (National Committee for Clinical Laboratory Standards, 1999). In brief, bacterial suspensions were diluted to 5.0 × 10^5^ colony-forming units (CFUs)/mL in fresh Mueller–Hinton broth. Drug concentrations of vancomycin or teicoplanin were adjusted to 1xMIC, 1/2xMIC, and 1/4xMIC. For each of four cephalosporins, the drug concentrations of 1- and 1/2-fold susceptible breakpoints were used for the combination with a glycopeptide. Bacterial counts were measured at 4, 8, and 24 h by enumerating the colonies in 10-fold serially diluted specimens of 100-μL aliquots plated on the nutrient agar (Difco Laboratories, Sparks, MD) at 37°C. All experiments were performed in duplicate.

Synergism was defined as a ≥2 log_10_ decrease in CFU/mL between the combination regimen and its most active constituent after 24 h, as well as the number of surviving organisms in the combination regimen, which must be ≥2 log_10_ CFU/mL below the starting inoculum. In addition, at least one of the combination drugs must be present at a concentration that does not affect the growth of the test organism (American Society for Microbiology, 2014). Bacteriostatic and bactericidal activities were defined as < 3 log_10_ and ≥3 log_10_ reductions in CFU/mL at 24 h, respectively, relative to the starting inoculum (National Committee for Clinical Laboratory Standards, 1999).

### MIC change ratios of glycopeptide MICs

The MICs of vancomycin or teicoplanin alone and combined with 1x or 1/2x susceptible breakpoint concentration (SBC) of a cephalosporin were determined by the agar dilution method. A MIC ratio indicates the fold of the MIC decline of the C-G combination vs. a glycopeptide alone.

### Statistical analysis

Data analyses were performed using SPSS for Windows 17.0 (SPSS Inc., Chicago, Illinois, USA). Because of the small sample sizes and violation of the normal distribution assumption of the OD ratio, the Mann–Whitney *U*-test was used to compare the differences between groups. The Kruskal–Wallis H and Dunn's tests were applied for multiple comparisons. The statistical significance was set to a *P* < 0.05.

## Results

### HCM and LCM MRSA isolates

**Figure 1:**
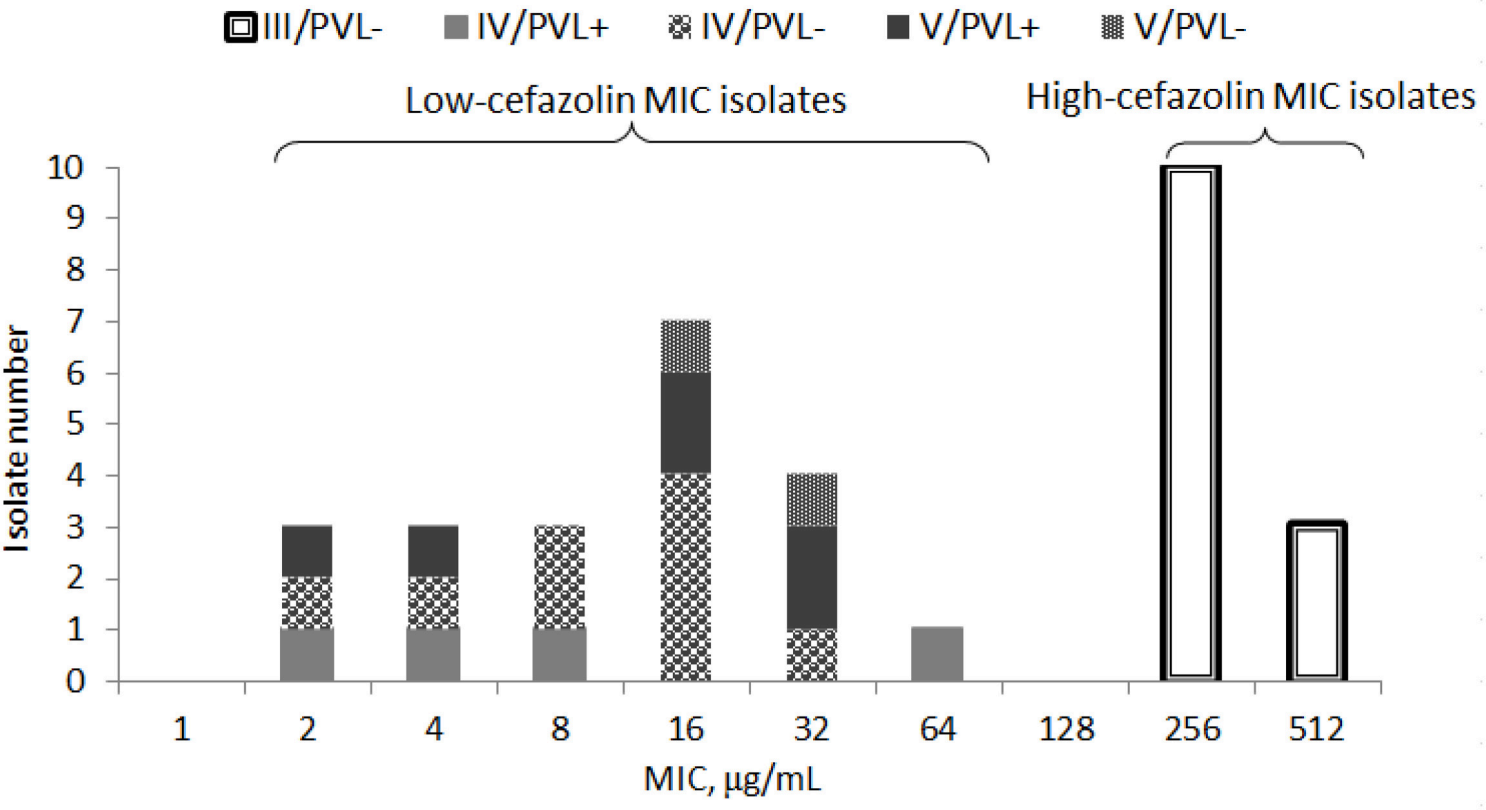
**Minimal inhibitory concentration (MIC) distribution of cefazolin in 34 isolates of methicillin-resistant *Staphylococcus aureus* stratified by SCC*mec* types (III, IV, or V) with or without Panton-Valentine leucocidin (PVL+ or PVL−)**.

**Figure 2:**
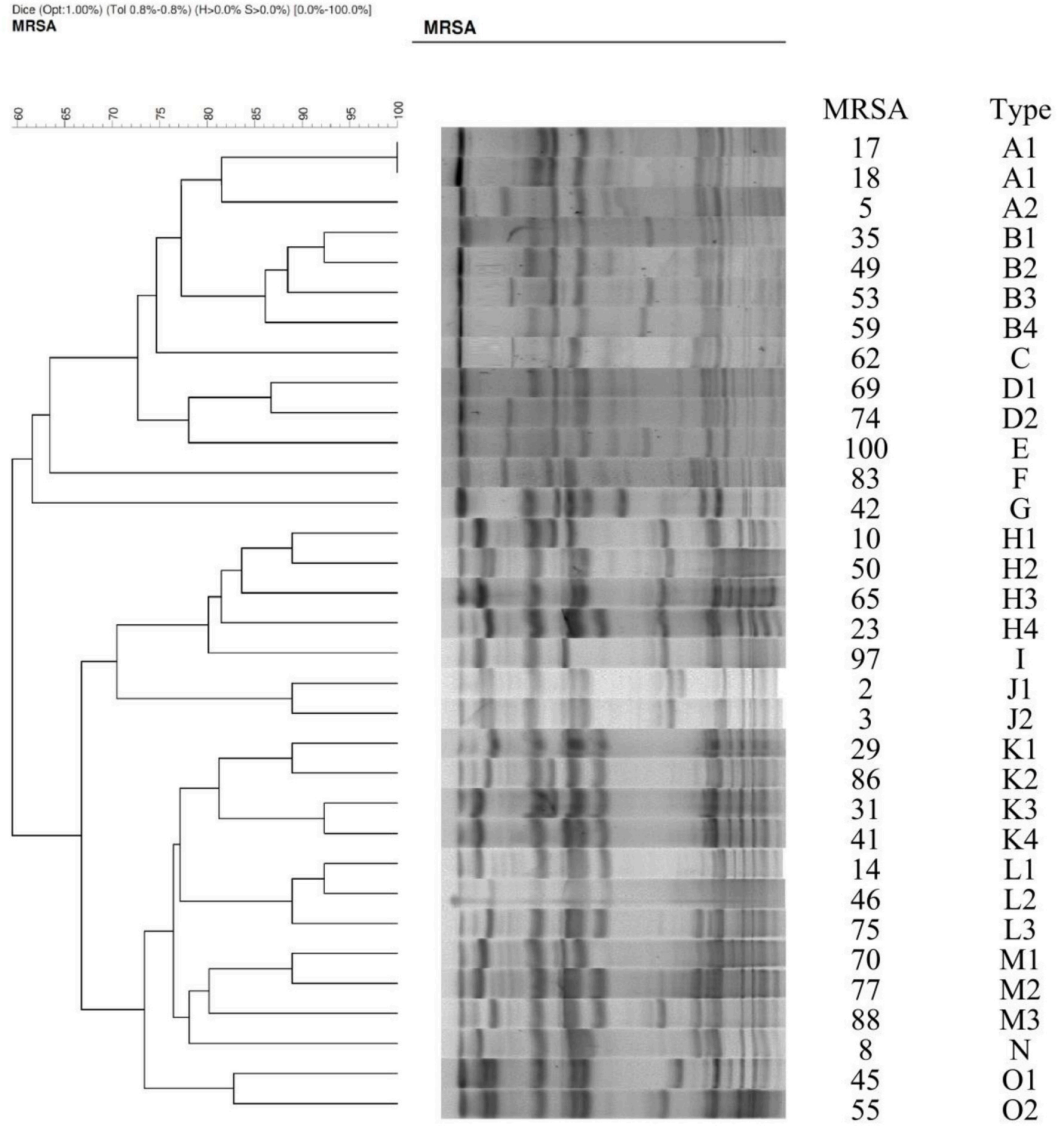
**PFGE profiles for 34 clinical isolates of methicillin-resistant *Staphylococcus aureus***.

Of 34 randomly selected MRSA isolates, 21 were LCM isolates, and 13 were HCM isolates. PVL genes were found in 10 isolates, which were LCM isolates. None of the HCM isolates harbored *lukF-PV* or *lukS-PV* (Figure 1). SCC*mec* typings of the LCM isolates included type IV (13 isolates) and V (8 isolates), and all 13 HCM isolates were type III. The pulsotype number of 13 HCM isolates was 6 (pulsotype A-F, one strain was untypeable), which was 10 for 21 LCM isolates (pulsotype G-P; Figure 2).

The MIC_90_ values of cefazolin, cefmetazole, cefotaxime and cefepime for HCM isolates (512, 512, 128, and 512 μg/mL, respectively) were higher than those of LCM isolates (32, 16, 64, and 64 μg/mL, respectively). A similar trend was also noted for oxacillin, minocycline, fusidic acid, ciprofloxacin, fosfomycin, and daptomycin. Of note, the MIC_90_s for HCM isolates was lower than for LCM isolates (16 vs. 128 μg/mL) for clindamycin. Additionally, for vancomycin or teicoplanin, the MIC ranges and MIC_90_ values were similar in the HCM and LCM isolates (Table 1).

**Table 1:** **Antimicrobial susceptibility of 18 antibiotics for methicillin-resistant *Staphylococcus aureus* with high- (MIC > 128 μg/mL) or low-cefazolin MICs (≤ 128 μg/mL)**.

**Antimicrobial agents**	**MIC, μg/mL**						**MIC breakpoints, μg/mL**		
	**High-cefazolin MIC isolates**, ***n*** = **13**			**Low-cefazolin MIC isolates**, ***n*** = **21**					
	**50%**	**90%**	**Range**	**50%**	**90%**	**Range**	**S**	**I**	**R**
Oxacillin	≥128	≥128	≥128	16	64	4~64	≤ 2	–	≥4
Cefazolin	256	512	256~512	16	32	2~128	≤ 8	16	≥32
Cefmetazole	64	128	32~128	8	16	4~16	≤ 16	32	≥64
Cefotaxime	512	512	512	32	64	8~64	≤ 8	16~32	≥64
Cefepime	512	512	256~512	32	64	8~128	≤ 8	16	≥32
Erythromycin	≥128	≥128	≥128	≥128	≥128	32~≥ 128	≤ 0.5	1~4	≥8
Gentamicin	≥128	≥128	≤ 1~≥ 128	≦1	≥128	≤ 1~≥ 128	≤ 4	8	≥16
Clindamycin	16	16	8~16	64	128	16~128	≤ 8	16	≥32
Tigecycline	0.5	0.5	0.25~0.5	0.5	0.5	0.25~1	≤ 0.5^a^	–	–
Minocycline	8	8	0.5~8	0.25	0.25	0.125~1	≤ 4	8	≥16
Fucidic acid	0.5	≥ 64	0.25~≥ 64	0.5	0.5	0.25~0.5	≤ 1^b^	–	–
Linezolid	4	4	2~4	4	4	2~8	≤ 4	–	≥8
Ciprofloxacin	≥64	≥64	64~≥ 64	0.25	1	0.25~≥ 64	≤ 1	2	≥4
Rifampin	0.016	0.016	0.016	0.016	0.031	0.016~0.031	≤ 1	2	≥4
Fosfomycin	16	32	2~32	4	8	1~16	≤ 64	128	≥256
Daptomycin	0.5	1	0.25~1	0.25	0.25	0.25~1	≤ 1	–	–
Teicoplanin	2	2	1~2	2	2	1~2	≤ 8	16	≥32
Vancomycin	2	2	1~2	1	2	1~2	≤ 2	4~8	≥16

a *breakpoint defined by FDA*.

b *breakpoint defined by BSAC*.

*MIC, minimal inhibitory concentration; S, susceptible; I, intermediate; R, resistant*.

### Checkboard method

With the checkerboard method, the FIC_90_ of vancomycin-based combinations ranged from 0.53 to 0.75, and teicoplanin-based combinations ranged from 0.51 to 0.62 in the HCM isolates (Table 2). Likewise, in the LCM isolates, the FIC_90_ of vancomycin-based combinations ranged from 0.51 to 0.56, and teicoplanin-based combinations ranged from 0.51 to 0.75. No antagonism was noted with either vancomycin or teicoplanin in combination.

**Table 2:** ***In vitro c*ombination effects of vancomycin (VAN) or teicoplanin (TEC) and one of the cephalosporins evaluated by the checkerboard method for 13 high-cefazolin MIC/low-cefazolin MIC isolates of methicillin-resistant *Staphylococcus aureus***.

**Drug combinations**	**Fractional inhibitory concentration (FIC)**			**Percentage, %**		
	**Range**	**50%**	**90%**	**Synergism**	**Indifference**	**Antagonism**
**VAN PLUS**						
Cefazolin	0.28~1/0.25~0.56	0.50/0.50	0.75/0.51	38.5/66.7	61.5/33.3	0/0
Cefmetazole	0.28~0.62/0.37~0.62	0.50/0.50	0.62/0.56	61.5/43.0	38.5/57.0	0/0
Cefotaxime	0.26~0.53/0.28~0.62	0.30/0.50	0.53/0.56	69.2/59.1	30.8/36.4	0/0
Cefepime	0.50~0.75/0.37~0.75	0.50/0.50	0.75/0.56	30.8/13.6	69.2/86.4	0/0
**TEC PLUS**						
Cefazolin	0.31~0.75/0.28~1	0.50/0.51	0.51/0.62	84.6/23.8	15.4/76.2	0/0
Cefmetazole	0.25~0.62/0.31~0.75	0.50/0.50	0.52/0.51	61.5/42.9	38.5/57.1	0/0
Cefotaxime	0.28~0.62/0.25~0.62	0.53/0.50	0.62/0.56	38.5/47.6	61.5/52.4	0/0
Cefepime	0.50~1/0.50~1	0.50/0.50	0.62/0.75	38.5/0.0	61.5/100.0	0/0

*MIC, minimal inhibitory concentration; synergism: FIC ≤ 0.5; indifference: 0.5 < FIC ≤ 4; and antagonism: FIC >4*.

Among vancomycin-based combinations, synergism was noted in 30.8% of the HCM isolates for cefepime and 69.2% for cefotaxime. In the LCM isolates, synergism was present in 13.6% for cefepime and 66.7% for cefazolin (Table 2). Among the teicoplanin-based combinations, 38.5% of the HCM isolates exhibited synergism that was lower for cefepime and cefotaxime, which was 84.6% for cefazolin. Synergism was not observed in any isolates for the cefepime-teicoplanin combination, but it was 47.6% for cefotaxime-teicoplanin combinations (Table 2).

With the checkerboard method, we found *in vitro* inhibitory activity of the combinations of 1/2x MIC of vancomycin or teicoplanin and 1/2x MIC of a cephalosporin in all 13 HCM isolates (Table 3). The inhibition effect can be observed in 9 of 13 HCM isolates even if cefazolin is used at a concentration of 1/128x MIC combined with vancomycin at a concentration of 1/2x MIC.

**Table 3:** **The inhibitory effect of vancomycin (VAN) or teicoplanin (TEC) in combination with one of four cephalosporins at a series of 2-fold dilution for 13 methicillin-resistant *Staphylococcus aureus* isolates with a high-cefazolin MIC**.

**VAN MIC**	**1/2x**	**1/4x**	**1/8x**	**1/16x**	**1/32x**	**1/64x**	**1/128x**	**1/256x**	**1/512x**
**CFZ MIC**									
1/2x	13	12	10	10	9	9	9	8	4
1/4x	10	5	2	1	1	0	0	0	0
1/8x	0	0	0	0	0	0	0	0	0
1/16x	0	0	0	0	0	0	0	0	0
**CMZ MIC**									
1/2x	13	13	13	11	9	8	5	5	1
1/4x	11	8	5	4	2	0	0	0	0
1/8x	5	1	0	0	0	0	0	0	0
1/16x	1	0	0	0	0	0	0	0	0
**CTX MIC**									
1/2x	13	13	13	13	13	11	10	9	8
1/4x	13	9	9	8	4	1	0	0	0
1/8x	8	0	0	0	0	0	0	0	0
1/16x	1	0	0	0	0	0	0	0	0
**CPM MIC**									
1/2x	13	13	9	7	6	6	6	5	2
1/4x	6	4	0	0	0	0	0	0	0
1/8x	1	0	0	0	0	0	0	0	0
1/16x	0	0	0	0	0	0	0	0	0
**TEC MIC**	**1/2x**	**1/4x**	**1/8x**	**1/16x**	**1/32x**	**1/64x**	**1/128x**	**1/256x**	**1/512x**
**CFZ MIC**									
1/2x	13	13	12	12	11	11	11	9	8
1/4x	11	11	4	1	0	0	0	0	0
1/8x	2	0	0	0	0	0	0	0	0
1/16x	0	0	0	0	0	0	0	0	0
**CMZ MIC**									
1/2x	13	13	12	11	11	11	7	3	0
1/4x	10	8	5	4	3	3	1	0	0
1/8x	2	0	0	0	0	0	0	0	0
1/16x	1	0	0	0	0	0	0	0	0
**CTX MIC**									
1/2x	13	13	13	10	8	6	4	4	3
1/4x	13	5	2	2	1	0	0	0	0
1/8x	7	0	0	0	0	0	0	0	0
1/16x	0	0	0	0	0	0	0	0	0
**CPM MIC**									
1/2x	13	12	12	10	9	9	7	6	6
1/4x	7	5	0	0	0	0	0	0	0
1/8x	2	0	0	0	0	0	0	0	0
1/16x	0	0	0	0	0	0	0	0	0

*MIC, minimal inhibitory concentration; CFZ, cefazolin; CMZ, cefmetazole; CTX, cefotaxime; CPM, cefepime. The numeric data indicate the isolate numbers with no visible growth overnight at different drug concentration combinations*.

### Time-killing method

In time-killing studies using 1x or 1/2x SBCs of a cephalosporin combined with 1/2x or 1/4x MIC of vancomycin or teicoplanin, bacterial loads are shown in Table 4. For a randomly selected HCM isolate, TIST-5, synergism was noted in any of four cephalosporins at a concentration of 1x SBC in combination with vancomycin at a concentration of 1/2x MIC, and there was a decline of 2.5–3.0 log_10_CFU/mL. At the concentration of 1/2x SBC, all cephalosporins, except cefotaxime, combined with vancomycin at the concentration of 1/2 MIC, leading to a bacterial load reduction of 2.8–3.6 log_10_ CFU/mL. The synergism was observed at combinations of 1/2x SBC cefmetazole and 1/4x MIC vancomycin with a colony count reduction of 3.12 log_10_ CFU/mL. For teicoplanin-based combinations, antibacterial activity was similarly potent, and the combination of 1/2x SBC cefmetazole and 1/4x MIC teicoplanin can cause a bacterial load reduction of 2.8 log_10_ CFU/mL.

**Table 4:** **Colony count changes at different concentrations of vancomycin or teicoplanin combined with a cephalosporin at the inhibitory or sub-inhibitory concentration**.

**Glycopeptide concentration**	**Control**	**Changes of colony count, log**_**10**_ **CFU/mL**							
		**CFZ**		**CMZ**		**CTX**		**CPM**	
		**8 μg/mL^a^**	**4 μg/mL^b^**	**16 μg/mL^a^**	**8 μg/mL^b^**	**8 μg/mL^a^**	**4 μg/mL^b^**	**8 μg/mL^a^**	**4 μg/mL^b^**
**TIST-5**									
Control	+3.10	+3.10	+3.10	+3.10	+3.10	+3.10	+3.10	+3.10	+3.10
VAN 1/2xMIC	+3.09	−3.00^s^	−2.75^c^	−3.00^s^	−3.60^s^	−2.49^c^	−1.94	−2.64^c^	−3.00^s^
VAN 1/4xMIC	+3.10	+3.10	+3.10	−1.64	−3.12^s^	+3.10	+3.10	+3.10	+3.10
TEC 1/2xMIC	+0.89	−2.70^c^	−2.82^c^	−3.12^s^	−2.90^c^	−2.49^c^	−0.96	−2.08^c^	−2.64^c^
TEC 1/4xMIC	+3.10	+0.92	+2.97	−3.60^s^	−2.82^c^	+2.72	+3.10	+2.98	+2.82
**TIST-10**									
Control	+3.08	+3.08	+3.08	+1.94	+3.08	+3.08	+3.08	+3.08	+3.08
VAN 1/2xMIC	+0.42	−2.39^c^	−2.30^c^	−2.66^c^	−2.36^c^	−2.47^c^	−2.30^c^	−2.36^c^	−2.44^c^
VAN 1/4xMIC	+3.08	−1.22	+0.01	−3.32^s^	−2.72^c^	+0.84	+3.08	+1.56	+3.08
TEC 1/2xMIC	+3.08	−3.92^s^	−2.47^c^	−2.17^c^	−3.02^s^	−2.32^c^	−2.28^c^	−2.05^c^	−2.20^c^
TEC 1/4xMIC	+3.08	−2.54^c^	−2.84^c^	−2.54^c^	−2.26^c^	−2.92^c^	+2.64	+0.04	+0.96

*MRSA, methicillin-resistant Staphylococcus aureus; MIC, minimal inhibitory concentration; CFZ, cefazolin; CMZ, cefmetazole; CTX, cefotaxime; CPM, cefepime*.

a *Susceptible MIC breakpoints for MRSA isolates*.

b *1/2 of susceptible MIC breakpoints for MRSA isolates*.

s *Bactericidal with synergistic effect*.

c *Bacteriostatic with synergistic effect*.

*TIST-5 (an isolate of high-cefazolin MIC) and TIST-10 (an isolate of low-cefazolin MIC) were randomly selected, and their bacterial load was measured at 24 h of co-cultivation with or without antibiotics*.

For another randomly selected LCM isolate, TIST-10, synergism was present in any of four cephalosporins at a concentration of 1x SBC combined with 1/2x MIC vancomycin or teicoplanin, which can cause bacterial load reductions of 2.4–2.7 and 2.1–2.5 log_10_ CFU/mL, respectively (Table 4). Moreover, a lower cephalosporin concentration, 1/2x SBC, in combination with 1/2x MIC vancomycin or teicoplanin can cause a bacterial load reduction of 2.3–2.44 or 2.2–2.5 log_10_ CFU/mL, respectively. When combined with 1/4x MIC vancomycin, 1x or 1/2x SBC cefmetazole can exhibit synergistic activity, resulting in a bacterial load reduction of 3.3 and 2.7 log_10_ CFU/mL, respectively, at 24 h of incubation. For teicoplanin-based regimens, 1/4x MIC teicoplanin can show synergism when combined with 1x or 1/2x SBC cefazolin or cefmetazole or with 1x SBC cefotaxime.

### Glycopeptide MICs with and without a cephalosporin

**Figure 3:**
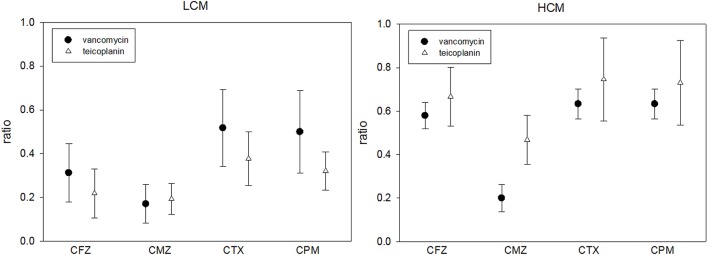
**The MIC ratios of vancomycin or teicoplanin in the presence and absence of 8 μg/mL cephalosporin in 21 low-cefazolin MIC (LCM) and 13 high-cefazolin (HCM) isolates**. (MIC ratio = glycopeptide MIC in the presence of 8 μg/mL of a cephalosporin/glycopeptide MIC without cephalosporin; means ± standard deviations).

The last way to examine the *in vitro* effect of C-G combinations is to evaluate the glycopeptide MIC change in the presence and absence of a cephalosporin. The MICs of vancomycin or teicoplanin were lower if there was any cephalosporin at the concentration of 1/2 SBC in culture media, which was independent of the cefazolin MIC. The MIC ratio of glycopeptides (glycopeptide MIC in the presence of a cephalosporin and glycopeptide) ranged from 1/6 to 3/4 (Table 5). If the cephalosporin concentration was fixed at 8 μg/mL, such changes in the glycopeptide MIC were most obvious with cefmetazole (Figure 3).

**Table 5:** **The MICs of vancomycin or teicoplanin in the absence and presence of a 1/2 susceptible breakpoint concentration of a cephalosporin for 13 high-cefazolin MIC and 21 low-cefazolin MIC isolates of methicillin-resistant *Staphylococcus aureus* (MRSA)**.

	**Control**	**CFZ, 4 μg/mL**	**CMZ, 8 μg/mL**	**CTX, 4 μg/mL**	**CPM, 4 μg/mL**
**VANCOMYCIN**					
**High-cefazolin MIC MRSA isolates**					
MIC range, μg/mL	0.75–1.5	0.5–0.75	0.25–0.5	0.5–1	0.5–1
Mean ±*SD*, μg/mL	1.54 ± 0.47	0.71 ± 0.09	0.48 ± 0.06	0.79 ± 0.14	0.75 ± 0.10
Mean fold of MIC decline	–	2.16	3.21	1.95	2.05
**Low-cefazolin MIC MRSA isolates**					
MIC range, μg/mL	0.75–1	0.25–0.5	0.125–0.5	0.5	0.25–0.5
Mean ±*SD*, μg/mL	0.99 ± 0.34	0.35 ± 0.13	0.15 ± 0.05	0.5 ± 0.00	0.48 ± 0.08
Mean fold of MIC decline	–	2.83	6.60	1.98	2.08
**TEICOPLANIN**					
**High-cefazolin MIC MRSA isolates**					
MIC range, μg/mL	0.75–1.5	0.5–0.75	0.125–0.5	0.5–1.25	0.5–1
Mean ±*SD*, μg/mL	1.38 ± 0.28	0.71 ± 0.09	0.25 ± 0.09	0.87 ± 0.22	0.81 ± 0.18
Mean fold of MIC decline	–	1.94	5.52	1.59	1.71
**Low-cefazolin MIC MRSA isolates**					
MIC range, μg/mL	0.75–1	0.125–0.5	0.125–0.25	0.25–0.75	0.25–0.75
Mean ±*SD*, μg/mL	0.79 ± 0.09	0.22 ± 0.10	0.15 ± 0.05	0.46 ± 0.12	0.45 ± 0.13
Mean fold of MIC decline	–	3.65	5.27	1.70	1.75

*MIC, minimal inhibitory concentration; CFZ, cefazolin; CMZ, cefmetazole; CTX, cefotaxime; CPM, cefepime; SD, standard deviation*.

## Discussion

According to our definitions of LCM and HCM, all 13 HCM isolates belonged to SCC*mec* type III and did not harbor PVL, and they were closely related to hospital-acquired MRSA (HA-MRSA) isolates (Sawanobori et al., 2015). On the other hand, the LCM isolates were SCC*mec* type IV or V and nearly half had PVL genes, which indicated genetic relatedness to community-acquired MRSA (CA-MRSA) isolates that have a higher prevalence of PVL (Liu et al., 2015). Therefore, the tested isolates somewhat represent the major types of clinical MRSA isolates.

Although the checkerboard method demonstrated synergism in < 50% of HCM or LCM isolates with several glycopeptide cephalosporin combinations, we found that the inhibitory effect of sub-inhibitory concentrations (1/2x MIC) of vancomycin or teicoplanin can be enhanced by low concentrations (1/512x MIC) of cephalosporin for HCM isolates. These results were also found in the time-killing study. Although the cephalosporin MIC-values of tested MRSA isolates were high (≥128 μg/mL), the antibacterial effect was evident based on a significant reduction of the glycopeptide MIC when serum achievable concentrations (1/2x or 1x SBC) of a cephalosporin were combined with sub-inhibitory concentrations of vancomycin or teicoplanin, as shown in Table 5. Such an *in vitro* effect of glycopeptide MIC reduction was most obvious for cefmetazole when the same drug concentrations of four cephalosporins were added (Figure 3). Therefore, although there were varied cephalosporin resistances in clinical MRSA isolates, there is solid evidence that cephalosporins can enhance the antibacterial activity of two commonly prescribed glycopeptides.

MSSA bacteremia should be treated by an anti-staphylococcal penicillin (nafcillin or oxacillin) or first generation cephalosporin (cefazolin) because several cohort studies reported poor clinical outcomes with vancomycin treatment (Chang et al., 2003; Kim et al., 2008; Schweizer et al., 2011). However, with the increasing prevalence of MRSA in the community and its presence in the healthcare facilities, patients with bacteremia due to Gram-positive cocci, should be treated with initial empirical therapy that includes an anti-MRSA agent until MRSA infection is excluded. Therefore, the combination of a first-generation cephalosporin and glycopeptide as an empiric regimen for Gram-positive coccus bacteremia not only covers MSSA, it enhances the antibacterial activity of glycopeptides for MRSA.

Although previous literature reported the benefit of C-G combinations for staphylococcal bacteremia (McConeghy et al., 2013), the concentrations of cephalosporin were relatively high. However, the concentrations we used in this study were susceptible to breakpoint concentration, which was relatively lower than the MIC of cephalosporins to MRSA. To the best of our knowledge, this is the first *in vitro* study with different methods to evaluate the C-G combinations, which exhibited enhanced antibacterial activity against clinical MRSA isolates, independent of the cephalosporin MIC.

A susceptible, but high, MIC (≥1 μg/mL by BMD or ≥1.5 μg/mL by *E*-test) to vancomycin is associated with increased mortality and treatment failure among patients with MRSA infections (Jacob and DiazGranados, 2013). Propensity score analysis demonstrated an increase in 28-day mortality as the vancomycin MIC increased from 0.75 to 3 μg/mL (*P* ≤ 0.001; Haque et al., 2010). For MRSA isolates with vancomycin MICs < 0.5 μg/mL, vancomycin was 55.6% successful in treating bacteremia, while vancomycin was only 9.5% effective in cases in which vancomycin MICs for MRSA were 1–2 μg/mL (Rahman et al., 2004). These results may be important in the era of increasing vancomycin MIC among clinical MRSA isolates for which vancomycin treatment failure was frequently encountered. Therefore, the MIC decrease of glycopeptides to MRSA may improve the clinical successful rate. These combinations may further reduce the MIC of vancomycin to MRSA. The application of C-G combinations, as one of feasible alternatives to treat MRSA infections, warrants further clinical investigation.

## Conclusion

Cephalosporin-glycopeptide combinations at clinically achievable concentrations can have *in vitro* synergistic antibacterial activities against a variety of clinical MRSA isolates. More animal experiments and clinical studies are required to validate their clinical utility in treating MRSA infections.

## Author contributions

HT is the guarantor of this manuscript; CL, CC, CZ, TW, WY, and HC contributed to the study concept and design; HZ, CC, YHC, and WK analyzed and interpreted the data; and HT, CC, WK, and YCC drafted the manuscript.

## Funding

This study was supported by a grant from the Ministry of Science and Technology of Taiwan (MOST 105-2314-B-384-007-MY3), Ministry of Health and Welfare (MOHW106-TDU-B-211-113003), and Chi-Mei Medical Center Research Foundation (CMFHT10501 and CMNCKU10509).

### Conflict of interest statement

The authors declare that the research was conducted in the absence of any commercial or financial relationships that could be construed as a potential conflict of interest.

## References

[B1] American Society for Microbiology (2014). Instructions to Authors. Washington, DC: American Society for Microbiology.

[B2] Andrews J. M. (2001). Determination of minimum inhibitory concentrations. J. Antimicrob. Chemother. 48(Suppl. 1), 5–16. 10.1093/jac/48.suppl_1.5 11420333

[B3] Carricajo A. Vermesch R. Aubert G. (2001). *In vitro* activity of cefpirome and vancomycin in combination against gentamicin-susceptible and gentamicin-resistant *Staphylococcus aureus*. Clin. Microbiol. Infect. 7, 218–226. 10.1046/j.1198-743x.2001.00238.x 11422245

[B4] Chang F. Y. Peacock J. E. Jr. Musher D. M. Triplett P. MacDonald B. B. Mylotte J. M.. (2003). *Staphylococcus aureus* bacteremia: recurrence and the impact of antibiotic treatment in a prospective multicenter study. Medicine 82, 333–339. 10.1097/01.md.0000091184.93122.09 14530782

[B5] Chang W. Ma X. Gao P. Lv X. Lu H. Chen F. (2015). Vancomycin MIC creep in methicillin-resistant *Staphylococcus aureus* (MRSA) isolates from 2006 to 2010 in a hospital in China. Indian J. Med. Microbiol. 33, 262–266. 10.4103/0255-0857.148837 25865979

[B6] Climo M. W. Patron R. L. Archer G. L. (1999). Combinations of vancomycin and beta-lactams are synergistic against staphylococci with reduced susceptibilities to vancomycin. Antimicrob. Agents Chemother. 43, 1747–1753. 10390234 10.1128/aac.43.7.1747 PMC89355

[B7] Clinical and Laboratory Standards Institute (2012). Approved Standard M100-S22 Twenty-Second Informational Supplement. Wayne, PA: Clinical and Laboratory Standards Institute.

[B8] Hagihara M. Wiskirchen D. E. Kuti J. L. Nicolau D. P. (2012). *In vitro* pharmacodynamics of vancomycin and cefazolin alone and in combination against methicillin-resistant *Staphylococcus aureus*. Antimicrob. Agents Chemother. 56, 202–207. 10.1128/AAC.05473-11 22006007 PMC3256059

[B9] Haque N. Z. Zuniga L. C. Peyrani P. Reyes K. Lamerato L. Moore C. L. (2010). Relationship of vancomycin minimum inhibitory concentration to mortality in patients with methicillin-resistant *Staphylococcus aureus* hospital-acquired, ventilator-associated, or health-care-associated pneumonia. Chest 138, 1356–1362. 10.1378/chest.09-2453 20558550

[B10] Hawser S. P. Bouchillon S. K. Hoban D. J. Dowzicky M. Babinchak T. (2011). Rising incidence of *Staphylococcus aureus* with reduced susceptibility to vancomycin and susceptibility to antibiotics: a global analysis 2004-2009. Int. J. Antimicrob. Agents 37, 219–224. 10.1016/j.ijantimicag.2010.10.029 21239146

[B11] Hsueh P. R. (2008). Tigecycline *in-vitro* surveillance in Taiwan (TIST). Int. J. Antimicrob. Agents 32(Suppl. 3), S173. 10.1016/S0924-8579(08)70022-0 19013349

[B12] Jacob J. T. DiazGranados C. A. (2013). High vancomycin minimum inhibitory concentration and clinical outcomes in adults with methicillin-resistant *Staphylococcus aureus* infections: a meta-analysis. Int. J. Infect. Dis. 17, e93–e100. 10.1016/j.ijid.2012.08.005 23089040 PMC3780595

[B13] Khokhlova O. E. Hung W. C. Wan T. W. Iwao Y. Takano T. Higuchi W.. (2015). Healthcare- and community-associated methicillin-resistant *Staphylococcus aureus* (MRSA) and fatal pneumonia with pediatric deaths in Krasnoyarsk, Siberian Russia: unique mRSA's multiple virulence factors, genome, and stepwise evolution. PLoS ONE 10:e0128017. 10.1371/journal.pone.0128017 26047024 PMC4457420

[B14] Kim S. H. Kim K. H. Kim H. B. Kim N. J. Kim E. C. Oh M. D.. (2008). Outcome of vancomycin treatment in patients with methicillin-susceptible *Staphylococcus aureus* bacteremia. Antimicrob. Agents Chemother. 52, 192–197. 10.1128/AAC.00700-07 17984229 PMC2223910

[B15] Lai C. C. Chen C. C. Chuang Y. C. Tang H. J. (2017). Combination of cephalosporins with vancomycin or teicoplanin enhances antibacterial effect of glycopeptides against heterogeneous vancomycin-intermediate *Staphylococcus aureus* (hVISA) and VISA. Sci. Rep. 7:41758. 10.1038/srep41758 28139739 PMC5282487

[B16] Lai C. C. Chen C. C. Huang H. L. Chuang Y. C. Tang H. J. (2016). The role of doxycycline in the therapy of multidrug-resistant *E. coli* – an *in vitro* study. Sci Rep. 6:31964. 10.1038/srep31964 27534373 PMC4989187

[B17] Lai C. C. Chu C. C. Cheng A. Huang Y. T. Hsueh P. R. (2015). Correlation between antimicrobial consumption and incidence of health-care-associated infections due to methicillin-resistant *Staphylococcus aureus* and vancomycin-resistant enterococci at a university hospital in Taiwan from 2000 to 2010. J. Microbiol. Immunol. Infect. 48, 431–436. 10.1016/j.jmii.2013.10.008 24388582 PMC7105077

[B18] Liu C. Chen Z. J. Sun Z. Feng X. Zou M. Cao W.. (2015). Molecular characteristics and virulence factors in methicillin-susceptible, resistant, and heterogeneous vancomycin-intermediate *Staphylococcus aureus* from central-southern China. J. Microbiol. Immunol. Infect. 48, 490–496. 10.1016/j.jmii.2014.03.003 24767415

[B19] Lodise T. P. Jr. McKinnon P. S. Levine D. P. Rybak M. J. (2007). Impact of empirical-therapy selection on outcomes of intravenous drug users with infective endocarditis caused by methicillin-susceptible *Staphylococcus aureus*. Antimicrob. Agents Chemother. 51, 3731–3733. 10.1128/AAC.00101-07 17664322 PMC2043293

[B20] Lozniewski A. Lion C. Mory F. Weber M. (2001). *In vitro* synergy between cefepime and vancomycin against methicillin-susceptible and -resistant *Staphylococcus aureus* and Staphylococcus epidermidis. J. Antimicrob. Chemother. 47, 83–86. 10.1093/jac/47.1.83 11152435

[B21] McConeghy K. W. Bleasdale S. C. Rodvold K. A. (2013). The empirical combination of vancomycin and a beta-lactam for Staphylococcal bacteremia. Clin. Infect. Dis. 57, 1760–1765. 10.1093/cid/cit560 23985343

[B22] Mongkolrattanothai K. Aldag J. C. Mankin P. Gray B. M. (2009). Epidemiology of community-onset *Staphylococcus aureus* infections in pediatric patients: an experience at a Children's Hospital in central Illinois. BMC Infect. Dis. 9:112. 10.1186/1471-2334-9-112 19607683 PMC2722661

[B23] Montanaro L. Ravaioli S. Ruppitsch W. Campoccia D. Pietrocola G. Visai L.. (2016). Molecular characterization of a prevalent ribocluster of methicillin-sensitive *Staphylococcus aureus* from orthopedic implant infections. correspondence with MLST CC30. Front. Cell Infect. Microbiol. 6:8. 10.3389/fcimb.2016.00008 26909340 PMC4754407

[B24] National Committee for Clinical Laboratory Standards (1999). Methods for Determining Bactericidal Activity of Antimicrobial Agents. Wayne, PA.

[B25] Niveditha N. Sujatha S. (2015). Worrisome trends in rising minimum inhibitory concentration values of antibiotics against methicillin resistant *Staphylococcus aureus*- Insights from a tertiary care center, South India. Braz. J. Infect Dis. 19, 585–589. 10.1016/j.bjid.2015.08.005 26361841 PMC9425378

[B26] Perlroth J. Kuo M. Tan J. Bayer A. S. Miller L. G. (2008). Adjunctive use of rifampin for the treatment of *Staphylococcus aureus* infections: a systematic review of the literature. Arch. Intern. Med. 168, 805–819. 10.1001/archinte.168.8.805 18443255

[B27] Rahman M. Kuhn I. Olsson-Liljequist B. Mollby R. (2004). Evaluation of a scanner-assisted colorimetric MIC method for susceptibility testing of gram-negative fermentative bacteria. Appl. Environ. Microbiol. 70, 2398–2403. 10.1128/AEM.70.4.2398-2403.2004 15066837 PMC383167

[B28] Ray P. Gautam V. Singh R. (2011). Methicillin-resistant *Staphylococcus aureus* (MRSA) in developing and developed countries: implications and solutions. Region. Health Forum 15, 74–82. 10.4103/0972-5229.118409

[B29] Sawanobori E. Hung W. C. Takano T. Hachuda K. Horiuchi T. Higuchi W.. (2015). Emergence of Panton-Valentine leukocidin-positive ST59 methicillin-susceptible *Staphylococcus aureus* with high cytolytic peptide expression in association with community-acquired pediatric osteomyelitis complicated by pulmonary embolism. J. Microbiol. Immunol. Infect. 48, 565–573. 10.1016/j.jmii.2014.04.015 25070278

[B30] Schweizer M. L. Furuno J. P. Harris A. D. Johnson J. K. Shardell M. D. McGregor J. C.. (2011). Comparative effectiveness of nafcillin or cefazolin versus vancomycin in methicillin-susceptible *Staphylococcus aureus* bacteremia. BMC Infect. Dis. 11:279. 10.1186/1471-2334-11-279 22011388 PMC3206863

[B31] Tang H. J. Chen C. C. Cheng K. C. Toh H. S. Su B. A. Chiang S. R.. (2012). *In vitro* efficacy of fosfomycin-containing regimens against methicillin-resistant *Staphylococcus aureus* in biofilms. J. Antimicrob. Chemother. 67, 944–950. 10.1093/jac/dkr535 22258931

[B32] Tang H. J. Chen C. C. Cheng K. C. Wu K. Y. Lin Y. C. Zhang C. C.. (2013). *In vitro* efficacies and resistance profiles of rifampin-based combination regimens for biofilm-embedded methicillin-resistant *Staphylococcus aureus*. Antimicrob. Agents Chemother. 57, 5717–5720. 10.1128/AAC.01236-13 23959320 PMC3811318

[B33] Tang H. J. Chen C. C. Ko W. C. Yu W. L. Chiang S. R. Chuang Y. C. (2011). *In vitro* efficacy of antimicrobial agents against high-inoculum or biofilm-embedded meticillin-resistant *Staphylococcus aureus* with vancomycin minimal inhibitory concentrations equal to 2 mug/mL (VA2-MRSA). Int. J. Antimicrob. Agents 38, 46–51. 10.1016/j.ijantimicag.2011.02.013 21549575

[B34] Teh S. H. Chi C. Y. Lin P. C. Ho C. M. Chou C. H. Tsai C. T.. (2015). Management and outcome of adults with skin and soft tissue infection caused by methicillin-resistant *Staphylococcus aureus* in a tertiary hospital in central Taiwan. J. Microbiol. Immunol. Infect. 48, 497–503. 10.1016/j.jmii.2014.08.030 25446039

[B35] Tenover F. C. Arbeit R. D. Goering R. V. Mickelsen P. A. Murray B. E. Persing D. H.. (1995). Interpreting chromosomal DNA restriction patterns produced by pulsed-field gel electrophoresis: criteria for bacterial strain typing. J. Clin. Microbiol. 33, 2233–2239. 7494007 10.1128/jcm.33.9.2233-2239.1995 PMC228385

[B36] Toyokawa M. Asari S. Nishi I. Horikawa M. Tsukamoto H. Sunada A.. (2003). *In vitro* combined effects of cefozopran/teicoplanin and cefozopran/vancomycin on methicillin-resistant *Staphylococcus aureus*. J. Chemother. 15, 31–36. 10.1179/joc.2003.15.1.31 12678411

[B37] Zhang K. McClure J. A. Elsayed S. Louie T. Conly J. M. (2005). Novel multiplex PCR assay for characterization and concomitant subtyping of staphylococcal cassette chromosome mec types I to V in methicillin-resistant *Staphylococcus aureus*. J. Clin. Microbiol. 43, 5026–5033. 10.1128/JCM.43.10.5026-5033.2005 16207957 PMC1248471

